# Expression and Function of PPARs in Placenta

**DOI:** 10.1155/2013/256508

**Published:** 2013-02-12

**Authors:** Satoru Matsuda, Mayumi Kobayashi, Yasuko Kitagishi

**Affiliations:** Department of Environmental Health Science, Nara Women's University, Kita-Uoya Nishimachi, Nara 630-8506, Japan

## Abstract

Peroxisome proliferator-activated receptors (PPAR) are members of the superfamily of nuclear hormone receptors involved in embryonic development and differentiation of several tissues including placenta, which respond to specific ligands such as polyunsaturated fatty acids by altering gene expression. Three subtypes of this receptor have been discovered, each evolving to achieve different biological functions. The PPARs also control a variety of target genes involved in lipid homeostasis. Similar to other nuclear receptors, the transcriptional activity of PPARs is affected not only by ligand-stimulation but also by crosstalk with other molecules. For example, both PPARs and the RXRs are ligand-activated transcription factors that coordinately regulate gene expression. In addition, several mechanisms underlying negative regulation of gene expression by PPARs have been shown. It is suggested that PPARs are key messengers responsible for the translation of nutritional stimuli into changes in gene expression pathways for placental development.

## 1. Introduction

Peroxisome proliferator-activated receptors (PPARs) are a family of ligand activated transcription factors belonging to the nuclear hormone receptor superfamily, which mainly regulate the expression of target genes involved in lipid and energy metabolism [1–3]. Three PPAR isotypes have been identified in mammals termed PPAR*α*, PPAR*β*/*δ*, and PPAR*γ* [4, 5]. Each isotype is a product of a separate gene, and each one has a distinct tissue distribution relating to the distinct functions. The PPARs play key roles in the metabolic syndrome and overall health of organisms including regeneration of tissues, differentiation, lipid metabolism, and immune response [6]. From a nutritional viewpoint, the PPARs are of importance because of their ability to be activated by long chain fatty acids and their metabolites [7]. Therefore, the PPARs are recognized as candidates in order to improve metabolism and health through suitable diet. In addition, several evidences show the important role of PPARs in reproductive organs [8, 9]. PPAR*γ* expression has been found in the granulosa, theca, and luteal cells [10]. The PPAR*γ* may regulate the differentiation and proliferation of the ovarian cells, steroidogenesis, angiogenesis, and prostaglandin production [11], indicating that PPARs modulate the estrous cycle and pregnancy. Retinoic X receptor (RXR) is a functional partner of PPAR. RXR*α* and PPAR*γ* function potently in metabolic diseases and are both important targets for antidiabetic drugs. Coactivation of RXR*α* and PPAR*γ* is believed to synergize their effects on glucose and lipid metabolism [12]. The RXR*α* and PPAR*γ* are essential for mouse placentogenesis [13, 14]. PPAR*γ* is important for mouse placenta morphology [15]. In addition, PPARs have also been implicated in several aspects of early pregnancy development including implantation, placentation, and trophoblast differentiation [16–18]. Furthermore, PPAR*γ* and RXR*α* are essential for cytotrophoblast cell fusion into a syncytiotrophoblast, which is obligatory for placentation, and these expressions are deregulated in pathological placenta [19]. So, the PPARs may be a link between energy metabolism and reproduction, which is frequently associated with insulin resistance. This paper will focus on the evidences of PPARs functions in placenta. We will also highlight the effects of co-modulators such as RXRs with PPARs in the experimental models.

## 2. Expression and Characteristics of PPAR 

**Figure 1:**
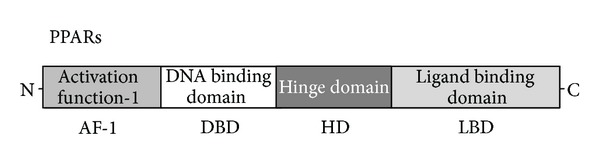
Schematic structure of PPARs protein. The predicted consensual important domain structures for each PPAR are depicted, which are common in fish species. AF-1 = activation function-1, DBD = DNA binding domain, HD = hinge domain linking DBD and LBD, LBD = C-terminal ligand binding domain.

PPARs (*α*, *β*, and *γ*) are nuclear hormone receptors that are known to regulate gene transcription and protein expression levels of fatty acid transport and metabolism mediating proteins through the formation of a DNA binding heterodimer complex [2–4]. All distinct PPAR subtypes share a high degree of structural homology with other members of the superfamily, particularly in the DNA-binding domain and ligand-binding domain (Figure 1). PPARs exhibit wide-ranging and isotype-specific tissue expression pattern [3–5]. PPAR*α* is expressed at high levels in tissues that catabolize fatty acids [20], as in the adult liver, heart, kidney, large intestine, and skeletal muscle. PPAR*β*/*δ* mRNA is ubiquitously distributed with a higher expression in the digestive tract and the placenta [21]. PPAR*γ* is mostly expressed in the adipose tissue [22] and immune system. The three isotypes are expressed as early as week 7 of gestation in endodermal and mesodermal origin cells [23]. There are limiting data describing the PPARs expression in endometrial tissue of animal species through the estrous cycle. PPAR*α* and PPAR*β* transcript levels show similar profiles during the estrous cycle [23–26]. PPAR*γ* mRNA level is quite stable during entire estrous cycle [24–26]. However, the precise role of PPARs in the uterus is not well known, although PPAR*α*, PPAR*β*, or PPAR*γ* expressions have been known in uterus of various species. High levels during the luteal phase and low during the follicular suggest the association with steroids function.

A wide variety of compounds have been identified as PPARs ligands. Among the synthetic ligands, fibrates and thiazolidinediones are PPAR*α* and PPAR*γ* agonists, respectively [27]. PPAR*γ* is also activated by prostaglandins and leukotrienes. In the presence of ligands, conformational changes of the ligand binding domain result in the recruitment of coactivator proteins, release of corepressor proteins, and subsequent assembly of a protein complex that enhances transcription of the target genes [28, 29]. A PPAR*α* specific ligand (8S-HETE), a PPAR*γ* ligand (15-deoxy-delta12, 14-prostaglandin J2), and a peroxisome proliferator (clofibrate) are all able to induce expression of both PPAR*α* and PPAR*γ* [30–32]. Subsequent work has led to the identification of various PPAR ligands that include eicosanoids, hypolipidemic agents, and antidiabetic drugs [33, 34]. 

Ligand activated PPARs bind as heterodimers with the RXRs on PPAR response elements. A number of PPAR target genes have been characterized to date. Most of these genes are known to have roles in lipid and glucose metabolism [35]. The endometrium is a possible place where PPARs may regulate cyclooxygenase- (COX-) 2 which catalyzes prostaglandin production [36]. They are critical to sustain the function of corpus luteum during the estrous cycle. The PPAR response element has been found upstream of the COX-2 transcriptional site. So, the activation of PPARs affects COX-2 expression in the epithelial cells. In COX-2 deficient mice, failures during the implantation and decidualization may be restored by administration of PPAR*β* agonists [37], suggesting common pathways of these molecules. Moreover, studies suggest that PPARs participate in uterine functions such as steroidogenesis, cytokine production, and angiogenesis during the estrous cycle and/or pregnancy [38, 39]. Interestingly, PPARs also downregulate nitric oxide synthase (NOS) in human cardiac myocytes and in human prostate cells [40]. As PPARs are expressed as cytotrophoblasts and syncytiotrophoblasts in the placenta, the activation of PPARs may stimulate the production and secretion of hormones such as gonadotropin required during pregnancy and fetal development [41, 42]. Thus, PPARs is essential for the maturation of a functional placenta.

## 3. Interaction with Retinoid X Receptor for Transactivation

**Figure 2:**
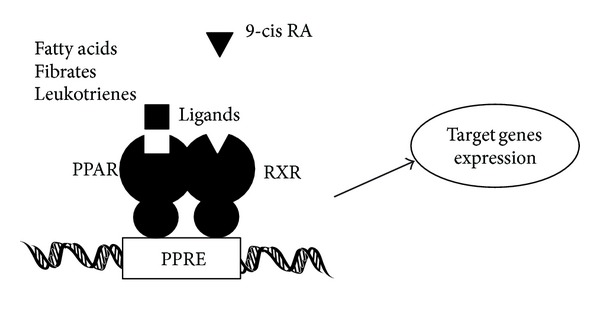
Schematic depiction of the model of mechanism of PPAR action. Similar to other nuclear hormone receptors, PPARs act as a ligand activated transcription factor. PPARs in response to the ligand binding heterodimerize with RXR and bind PPRE DNA sequences in the promoters of target genes, which are often involved in the lipid metabolism. Note that some critical molecules have been omitted for clarity.

PPARs bind to a variety of PPAR response elements (PPREs) present in the promoter regions of the responsive genes. The transcriptional regulation by PPARs requires heterodimerization with the retinoid X receptor (RXR) (Figure 2). Retinoic acid affects a broad spectrum of physiological processes, including cell growth, differentiation, morphogenesis, reproduction, and development [43], through the action of two types of receptors, the retinoic acid receptors (RARs) and the retinoid-X-receptors (RXRs). When activated by a ligand, the heterodimer modulates transcription activity. The transcriptional control by the PPAR/RXR heterodimer also requires interaction with coregulator complexes [44]. Thus, selective action of PPARs in vivo results from the interplay at a time point of each of the cofactors available. The RXRs are able to influence the transcription of a wide variety of genes, because they can activate gene transcription by binding to specific sites on DNA as homodimers and/or as the heterodimers with other related nuclear receptors including the PPARs, vitamin D receptor, and thyroid hormone receptors [45–47]. The temporal and spatial patterns of expression of PPARs and RXRs isoforms in the developing placenta have been elucidated [48]. In the human placenta, PPAR*α*, PPAR*β*, and PPAR*γ* are observed, while RXR*β* is not detected. Immunocytochemistry staining results also determine the presence of PPAR*α*, PPAR*β*, PPAR*γ*, RXR*α*, and RXR*γ* to be specific to the trophoblast layer of the human chorionic villi [49]. The presence of PPAR and RXR isoforms in placenta suggests that PPAR and RXR isoforms are potential regulators of placental lipid transfer and homeostasis. The PPARs/RXRs heterodimers may play a key regulatory role in placental development. It has been suggested that the PPARs/RXRs heterodimers may also function in the modulation of trophoblast invasion. There was no significant difference in PPARs or RXRs protein expression in both amnion and choriodecidua, which have been identified in gestational tissues [50–52]. 

## 4. Functional Interplay for the Transrepression of PPARs

**Figure 3:**
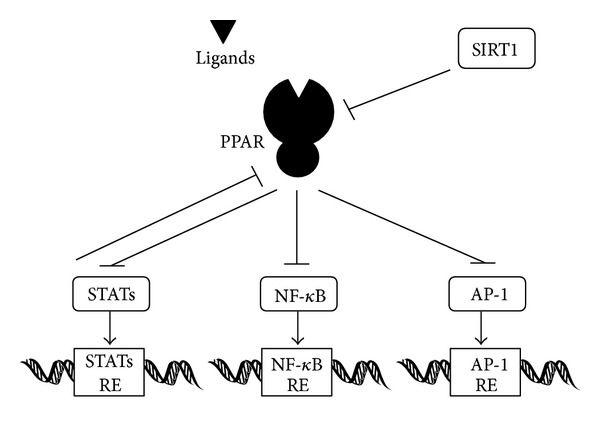
A hypothetical schematic implication of some of the PPAR regulatory network. Examples of molecules known to interact with PPARs pathway in mammals are shown. Hammerheads mean inhibition. Note that some critical pathways have been omitted for clarity.

The NAD(+)-dependent histone deacetylase Sir2 regulates life-span in various species [53]. Mammalian homologs of Sir2 are called Sirtuins (SIRT1–SIRT7) [54]. PPAR*α* and SIRT1 coordinately suppress genes involved in mitochondrial function [55] (Figure 3). Calorie restriction extends lifespan in organisms ranging from yeast to mammals. Upon food withdrawal, SIRT1 protein binds to and represses genes controlled by the fat regulator PPAR*γ*, including genes mediating fat storage. SIRT1 represses PPAR*γ* by docking with its cofactors nuclear receptor corepressor and silencing mediator of retinoid and thyroid hormone receptors [56, 57]. The repression of PPAR*γ* transactivation by SIRT1 inhibits lipid accumulation in adipocytes. SIRT1 also regulates angiogenesis signaling [58], which is expressed in the vasculature during blood vessel growth. Loss of SIRT1 function blocks sprouting angiogenesis and branching morphogenesis of endothelial cells with consequent downregulation of genes involved in blood vessel development and vascular remodeling. Human SIRT1 and SIRT2 are localized in the syncytiotrophoblast layer and the cytotrophoblasts of the placenta, amnion epithelium, trophoblast layer of the chorion, and decidual cells [59]. Resveratrol decreases proinflammatory TNF, IL6, and IL8 gene expression and resultant prostaglandin release from the gestational tissues [59]. SIRT1 also modulates gene expression in target tissues by regulating transcriptional coregulators or by directly interacting with transcription factors. SIRT1 overexpression prevents cytokine-mediated cytotoxicity, nitric oxide (NO) production, and inducible NO synthase expression. PPARs and SIRT1 may play a pivotal role in regulating pregnancy and parturition [59]. 

Many of the anti-inflammatory effects of PPAR*γ* are caused by antagonizing the activities of the transcription factors including nuclear factor-kappa B (NF-*κ*B) (Figure 3). EPA inhibited the NF-*κ*B pathway in myotubes in a PPAR*γ*-dependent manner. In one way for the inhibition, PPARs and these transcription factors bind each other via protein-protein interactions and prevent binding to their response elements. The ligand-activated PPARs have been shown to interfere with DNA binding of both AP-1 and NF-*κ*B activity [60]. Furthermore, the mitogen-activated protein kinase (MAPK) pathway is also regulated by PPARs at different levels [61]. In addition, activation of PPAR*γ* reduces c-Jun N-terminal kinase (JNK) and p38 MAPK activation, leading to downregulation of proinflammatory gene expression [62]. The transcription factors NF*κ*B, CCAAT/enhancer-binding protein (CEBP), and AP-1 are important transcription factor families that are involved in immune and inflammatory functions as well as in cell growth and differentiation. Human placenta is rich in diverse bioactive molecules, whose extract induces interleukin mRNA and protein expressions in a dose-dependent manner. For example, the IL8 promoter contains binding sites for the NF*κ*B, AP-1, and CEBP. The IL-8 expression is inhibited by an inhibitor of JNK [63, 64]. 

Interestingly, the transcriptional expression levels of fatty acid binding proteins are upregulated in males and downregulated in females [65]. A similar trend between sexes occurs for PPARs and CEBPs, which may be the upstream regulatory elements [65]. Estrogen-related receptors have been identified as PPAR coactivators, which upregulate the expression of PPAR*α* and PPAR*α*-regulated genes. Estrogen has not been reported to be a PPAR ligand, but interactions between PPARs and ER proteins and their response elements have been described [66]. These interactions might be due to estrogen induced production of PPAR activating metabolites. Studies have found that 17beta-estradiol upregulates the expression of PPAR*α* in skeletal muscle of rats [67].

A direct relationship between the PPARs activation and the inhibition of STAT5 mediated transcription has been reported [68, 69]. The PPARs do not block STAT5 tyrosine phosphorylation or do not inhibit DNA-binding activity but inhibit the transcriptional activity of STAT5. Conversely, activated STAT5 is able to inhibit PPAR-regulated gene transcription. In other words, STAT5-activating hormones and cytokines may modulate the responsiveness of PPARs to the chemical ligands. The cross-inhibition between PPAR and STAT5 proceeds in a synchronized and bidirectional manner (Figure 3). Exposure to environmental chemical activators of PPARs may thus lead to alteration of hormone induced STAT5-regulated gene expression in tissues such as placenta, where both transcription factors are expressed.

## 5. Perspective

PPARs are lipid-activated transcription factors that have emerged as key regulators of both lipid metabolism and inflammation, and they exert positive and negative controls over the expression of a range of genes. However, the range of transcription factors affected and the molecular mechanism involved may be different for each PPAR isoforms and cell types. Furthermore, peroxisome proliferators induce numerous alterations in lipid metabolism. A comparative approach to bring together physiological and nutritional roles of PPARs across species appears critical. It is now clear that PPARs are important in the control of placental development. PPARs may play a key role in linking lipid metabolism and reproduction systems. In addition, the PPAR*γ*/RXR*α* signaling is important in human cytotrophoblast and cell fusions. A disturbed PPAR*γ*/RXR*α* pathway could contribute to pathological human pregnancies. SIRT1 expression is downregulated by proinflammatory cytokines. Possessing anti-inflammatory action in human gestational tissues, the SIRT1 expression is downregulated by proinflammatory cytokines. The natural polyphenol resveratrol inhibits cytokine and prostaglandin release via the SIRT1 activation. Both mRNA and protein levels of SIRT1 are shown to decrease in placenta and fetal membranes after labor onset, which may contribute to uterine contractions associated with labor. It would be of interest to investigate the impact of different types of fatty acid, integrated into food, on ovulation capacity and fetal development. Further study of PPARs, RXRs, and SIRTs functions in placenta may indicate pathways that are common to critical processes, providing additional focus for research in important human placental diseases. In parallel, defining more specific mode of action by identifying the endogenous coactivators and modulators of these transcription factors in animal models will help to build more efficient therapeutic strategy for the diseases. Future studies using functional genomic approaches will be required to more clearly establish the complicated mechanisms by which PPARs exert their actions. Additional insight is also needed into endogenous PPAR and RXR ligands, how these molecules are formed, and how they are delivered to the nucleus in placenta. Furthermore, additional experiments are required to increase the knowledge of the way in which lipid metabolism influences reproductive functions.

## Abbreviations

AP-1: Activator protein-1
CEBP: CCAAT/enhancer-binding protein
COX: Cyclooxygenase
JNK: c-Jun N-terminal kinase
MAPK: Mitogen activated protein kinase
NAD(+): Nicotinamide adenine dinucleotide
NF-*κ*B: Nuclear factor-kappa B
NO: Nitric oxide
PPAR: Peroxisome proliferator-activated receptor
PPRE: PPAR response element
RA: Retinoic acid
RE: Response element
RXR: Retinoid X receptor
SIRT1: Silent mating type information regulation 2 homolog 1
STAT: Signal transducers and activators of transcription.
